# Streamlined cell-free protein synthesis using wheat germ extract

**DOI:** 10.1007/s00253-026-13914-x

**Published:** 2026-06-11

**Authors:** Zsuzsanna Szeitner, Alexandra Krisztina Tar, Ákos Harkai, Anna Gyurkovics, Tamás Mészáros, Brigitta Margit Kállai

**Affiliations:** Department of Molecular Biology, Semmelweis University, Budapest, Hungary

**Keywords:** Cell-free protein synthesis, Eukaryotic proteins, Coupled in vitro transcription–translation, Wheat germ extract

## Abstract

**Abstract:**

Wheat germ extract (WGE)-based cell‑free translation is a robust and versatile platform for small‑scale, high‑throughput production of diverse eukaryotic proteins. Although the system has been optimised for efficient protein synthesis using cap‑independent translation‑compatible vectors and bilayer in vitro translation formats, it has traditionally relied on separate transcription and translation steps. To streamline this workflow, a bilayer, coupled in vitro transcription–translation (cIVTT) protocol was developed in which both processes are integrated into a single reaction. After optimisation of the reaction mixture composition and reaction conditions, protein yields comparable to those obtained with the conventional two‑step bilayer in vitro translation method were achieved. Notably, the cIVTT protocol remained effective at reduced temperatures, thereby supporting the enhanced production of human cardiac muscle Troponin I (cTnI), a protein with demanding folding requirements. This simplified and time‑efficient approach enables microgram‑scale protein production and is well suited for high‑throughput applications.

**Key points:**

*• Streamlined WGE cell-free protein synthesis workflow*

*• No plasmid linearisation needed before coupled in vitro transcription/translation*

*• Soluble eukaryotic protein yields on par with two‑step bilayer format*

**Supplementary Information:**

The online version contains supplementary material available at 10.1007/s00253-026-13914-x.

## Introduction

Advancements in prokaryotic and eukaryotic cell-based protein synthesis systems have greatly enhanced routine protein production. However, significant challenges remain in the high-throughput production of eukaryotic proteins in their correctly folded, native conformations. Eukaryotic cell-based in vitro translation systems—characterised by their translational flexibility and rapid synthesis capabilities—represent a rational alternative for fast and efficient protein production (Hunt et al. 2024).

Among the diverse cell-free protein synthesis (CFPS) systems derived from various organisms, wheat germ extract (WGE)-based translation platforms have gained significant attention (Kállai et al. 2025). This is largely due to their relatively low sensitivity to codon bias, high efficiency in correctly folding eukaryotic proteins, and their capacity to synthesise protein complexes (Banko et al. 2024; Fogeron et al. 2021). Notwithstanding these advantages, a notable bottleneck of most existing WGE translation protocols is their reliance on a two-phase process, where transcription and translation occur separately. Performing the two steps separately requires additional handling, which increases overall turnaround time and constrains high-throughput application. Moreover, this approach introduces a risk of mRNA degradation during storage and transfer, potentially leading to reduced translational efficiency.

Although coupled in vitro transcription–translation (cIVTT) workflows are inherently simpler than the conventional two‑step transcription–translation approach—and DNA‑driven WGE cIVTT kits were already commercially available by the mid‑1990s—the mRNA‑programmed, two‑step format remains the mainstay of WGE CFPS. Nevertheless, to reduce turnaround time and simplify handling, WGE cIVTT platforms have been developed. For example, Zhao et al. (2007) reported an SP6‑driven vector that leverages the barley yellow dwarf virus (BYDV) IRES for cap‑independent translation initiation, achieving dialysis/CECF (continuous-exchange cell-free) yields comparable to those of two‑step WGE protocols. More recently, both transcriptional and translational regulatory elements have been re‑engineered to establish a new WGE cIVTT system (Ogawa and Takamatsu 2019). The SP6 promoter was replaced with a T7 promoter, which operates efficiently across a broader temperature range and is therefore expected to perform better at the lower temperatures optimal for WGE-based translation (Chamberlin and Ring 1973). The translation enhancer used in this system is a mutated variant of the cricket paralysis virus (CrPV) IRES, previously shown to function effectively in WGE (Ogawa and Takamatsu 2019). When linear, PCR‑generated DNA templates were used, this cIVTT system outperformed conventional mRNA‑addition‑based translation in batch format (Takahashi and Ogawa 2021).

We overcame the limitations of two‑step CFPS by developing a bilayer cIVTT protocol that uses a commercially available WGE‑based cell‑free system and builds on previously reported methodologies. In this approach, we used T7 RNA polymerase (RNAP)—which remains highly active and robust even under suboptimal temperature conditions—together with the T7 promoter‑driven, WGE‑compatible pEU3 vector family (Bardóczy et al. 2008; Nagy et al. 2020). Because nucleoside triphosphates (NTPs) sequester Mg^2^⁺ and can reduce translational efficiency in cIVTT, we supplemented the reaction with equimolar MgCl₂ to restore efficient translation (Takahashi and Ogawa 2021). After optimising DNA template concentrations, we showed that the cIVTT system operates efficiently at 20 °C, a temperature that favours expression of difficult‑to‑fold proteins. By integrating mRNA synthesis and protein translation into a single bilayer reaction, our protocol streamlines protein production and suits high‑throughput WGE‑based translation workflows.

## Materials and methods

### Preparation of DNA template

The pEU3-NII_tGFP (Turbo green fluorescent protein derived from *Pontellina plumata*, FPbase ID: A315M with C-terminal 6xHis-tag), pEU3-NII-GLICNot_CDH11 (Uniprot: P55287, human Cadherin-11, amino acids 54–⁠383 representing EC1–⁠EC3 extracellular domains), pEU3-NII-GLICNot_RCE2 (Uniprot: Q9ZU75, *Arabidopsis thaliana* RUB1-conjugating enzyme 2), and pEU3-NII-GLICNot_TNNI3 (Uniprot: P19429, human cardiac muscle Troponin I, cTnI) plasmids were constructed as described previously (Nagy and Mészáros 2014) without codon optimisation of the protein coding sequences (provided in Supplementary Fig. S1). Plasmid DNA was isolated using NucleoBond Xtra Midi kit (Macherey-Nagel, Düren, Germany, #740412.50) according to the manufacturer’s instructions. pEU3-NII_tGFP plasmid was linearised by digestion with the restriction enzyme *Sca*I, whereas pEU3-NII-GLICNot_CDH11, pEU3-NII-GLICNot_RCE2, and pEU3-NII-GLICNot_TNNI3 plasmids were linearised using *Not*I. Restriction digestion reactions were performed using 3 µg of plasmid DNA, 3 µL of 10 × FastDigest restriction buffer, and 3 µL of the corresponding FastDigest restriction enzyme (Thermo Scientific, Waltham, MA, USA), with nuclease-free water added to a final reaction volume of 30 µL. Digestion reactions were incubated at 37 °C for 2 h and inactivated by heat. Complete linearisation and DNA integrity were confirmed by agarose gel electrophoresis.

Linearised DNA samples were precipitated with polyethylene glycol (PEG) (Rosenthal et al. 1993). Purified DNA was resuspended in 10 µL of nuclease-free distilled water. The concentration was measured by NanoDrop One (Thermo Scientific, Waltham, MA, USA) using dsDNA programme (target concentration ≥ 230 ng/µL), and the sample stored at − 20 °C until further use.

### Optimisation of coupled in vitro transcription–translation (cIVTT)

Small-scale cIVTT reactions were performed using the WGE CFPS system of CellFree Sciences (Matsuyama, Japan). Fifteen millimolar MgCl₂/NTP mix was prepared in nuclease-free water to contain 15 mM MgCl₂ and 15 mM NTPs in total (ATP, GTP, CTP, and UTP at 3.75 mM each), using 50 mM MgCl₂ (Bio-Rad Laboratories, Hercules, CA, USA, #172–5300) and 100 mM Tris-buffered NTPs (Thermo Scientific, Waltham, MA, USA; TranscriptAid T7 High Yield Transcription Kit, #K0441), respectively, as stock solutions. The T7 RNAP (Thermo Scientific, Waltham, MA, USA) stock solution was diluted 1:5 in nuclease-free water immediately before use. The translation mixture was assembled in 5 µL volume by combining the following components: 2.5 µL WEPRO7240 WGE (CellFree Sciences, Matsuyama, Japan), 300 ng linearised or circular DNA template, 0.2 µL 1 mg/mL creatine kinase solution (Roche, Basel, Switzerland, #10127566001), 0.5 µL 15 mM MgCl₂/NTP mix, and 0.5 µL 1:5 diluted T7 RNAP. The reaction volume was adjusted to 5 µL with nuclease-free water, if needed. Components were mixed by gentle pipetting, taking care to avoid bubble formation. The feeding solution was pre-mixed in a PCR tube from 1 × SUB-AMIX SGC (CellFree Sciences, Matsuyama, Japan; diluted from 40 × S1, S2, S3, S4 stock solutions in nuclease-free water) supplemented with 15 mM MgCl₂/NTP mix (prepared as described above) to 1.5 mM final concentration. The 5 µL translation mixture was carefully underlaid beneath 51.5 µL of feeding solution in a PCR tube, ensuring that the two layers remained separate. For the batch reactions, the two layers were deliberately mixed at the start of incubation. The PCR tube was sealed and incubated at the indicated temperature (20 °C as default) for the indicated length of time (20 h as default). Following incubation, the reaction mixture was gently mixed and collected, and then stored on ice until downstream analysis.

The conventional bilayer two-step in vitro transcription followed by translation was set up according to a previously described protocol with slight modifications (Nagy et al. 2020). Briefly, the linearised plasmids were used as templates of in vitro transcription reactions with TranscriptAid T7 High Yield Transcription Kit according to the manufacturer’s protocol. 5.2 µL translation mixture containing 2.5 µL W7240 WGE, 1.875 µL 1 × SUB-AMIX SGC, 0.625 µL crude mRNA, 0.2 µL 1 mg/mL creatine kinase was gently mixed and carefully underlaid to 51.5 µL 1 × SUB-AMIX SGC solution in a PCR tube. The two-step translation reactions were carried out for 20 h at 15 °C or 20 °C.

### Fluorescence measurements of green fluorescent protein

After incubation, translation mixtures were homogenised and transferred into a black 384-well plate (Greiner, Kremsmünster, Austria, #784076, F-bottom, small volume). Fluorescence spectra of translated tGFP were acquired at 25 °C using a CLARIOstar plate reader (BMG Labtech, Ortenberg, Germany) in fluorescence spectrum mode, with excitation scanned from 400 to 510 nm and emission scanned from 464 to 600 nm (Supplementary Fig. S2). The fluorescence intensities of translated tGFP were also measured at 25 °C by CLARIOstar system using 470/515 nm excitation/emission wavelengths. Fold changes were calculated per biological replicate to enable comparison between experiments and analysed using one-way or two-way ANOVA, followed by Tukey’s multiple comparisons test (GraphPad Prism v.11.0.0).

### Western blot analysis of synthesised proteins

After incubation for the indicated length of time (20 h as default), 55 µL translation mixture was spun down at 15,000 g for 15 min, supernatant was collected and transferred in an Eppendorf tube, and the precipitate was resuspended in 55 µL PBS. Western blot analysis of different glutathione S-transferase (GST)-tagged proteins was performed. In brief, 3 µL protein samples were mixed with SDS-DTT sample buffer and heated for 5 min at 95 °C. The protein samples were separated on 10% or 15% SDS–PAGE with Laemmli buffer at constant 20 or 23 mA current (with voltage set to maximum 200 V) and transferred to low fluorescence polyvinylidene fluoride (PVDF) membrane (Merck Millipore, Darmstadt, Germany) by wet blotting using Dunn’s carbonate buffer (10 mM NaHCO_3_, 3 mM Na_2_CO_3_, 10% methanol, pH 9.9) at constant 30 V voltage, overnight at 4 °C (Dunn 1986) or semi-dry blotting using Trans-Blot Turbo Transfer System (Bio-Rad Laboratories, Hercules, CA, USA, #1704272). Following transfer, the membranes were air-dried, reactivated with methanol, washed with phosphate buffered saline (PBS), and blocked for 60 min in Intercept (PBS) Blocking Buffer (LI-COR Biosciences, Bad Homburg, Germany, #927–70001). Blots were incubated overnight at 4 °C with rabbit anti-GST primary antibodies (Upstate, Merck KGaA, Darmstadt, Germany, clone 06–332) or mouse anti-polyHis primary antibodies (Sigma-Aldrich, St. Louis, MO, USA, clone HIS-1, #H1029) diluted 1:2000 in Intercept (PBS) Blocking Buffer with 0.2% Tween 20, washed four times 5 min each in PBS supplemented with 0.1% Tween 20 and incubated for 1 h at room temperature with IRDye® 680RD or 800CW goat anti-rabbit (LI-COR Biosciences, Bad Homburg, Germany, #926–68071/#926–32211) or IRDye® 680RD goat anti-mouse (LI-COR Biosciences, Bad Homburg, Germany, #926–68070) (1:20,000 dilution in PBS-based Intercept Blocking Buffer supplemented with 0.01% SDS and 0.2% Tween 20). After the washing step, the image was taken by Odyssey CLx (LI-COR Biosciences, Bad Homburg, Germany) instrument using red and green channels. Band intensities were quantified by densitometric analysis using Image Studio Ver 6.1 and analysed using one-way or two-way ANOVA, followed by Tukey’s multiple comparisons test (GraphPad Prism v.11.0.0). Uncropped western blot images are provided in the Supplementary Material.

## Results

### Assessing the functionality of the bilayer cIVTT system

In cIVTT, the optimal conditions for two distinct processes, i.e. RNA polymerisation and protein synthesis must be accommodated within a single reaction buffer. It has been well documented that magnesium ions are essential for ribosomal assembly and structural stability and are therefore critical for efficient protein synthesis (Fogeron et al. 2021). During cIVTT, however, the high concentrations of NTPs required for RNA synthesis can sequester Mg^2^⁺ ions, thereby reducing their availability and limiting translational efficiency (Jewett and Swartz 2004). This inhibitory effect on translation can be alleviated by supplementing the reaction with Mg^2^⁺ at concentrations equimolar to the NTPs (Ma and Wang 2016). Accordingly, the in vitro translation mixture was supplemented with balanced concentrations of 1.5 mM Mg^2^⁺ and 1.5 mM NTPs (0.375 mM each nucleotide), T7 RNAP, and varying amounts of DNA template to check the applicability of this approach. The 1 × SUB-AMIX SGC feeding buffer was also supplemented with the same concentrations of Mg^2^⁺ and NTPs. We did not separately optimise the T7 RNAP concentration because the activity of the proprietary enzyme was not disclosed. In a preliminary experiment (data not shown), a slightly decreased tGFP yield was observed with concentrated T7 RNAP relative to the 1:5 dilution; therefore, the diluted enzyme was used.

**Fig. 1:**
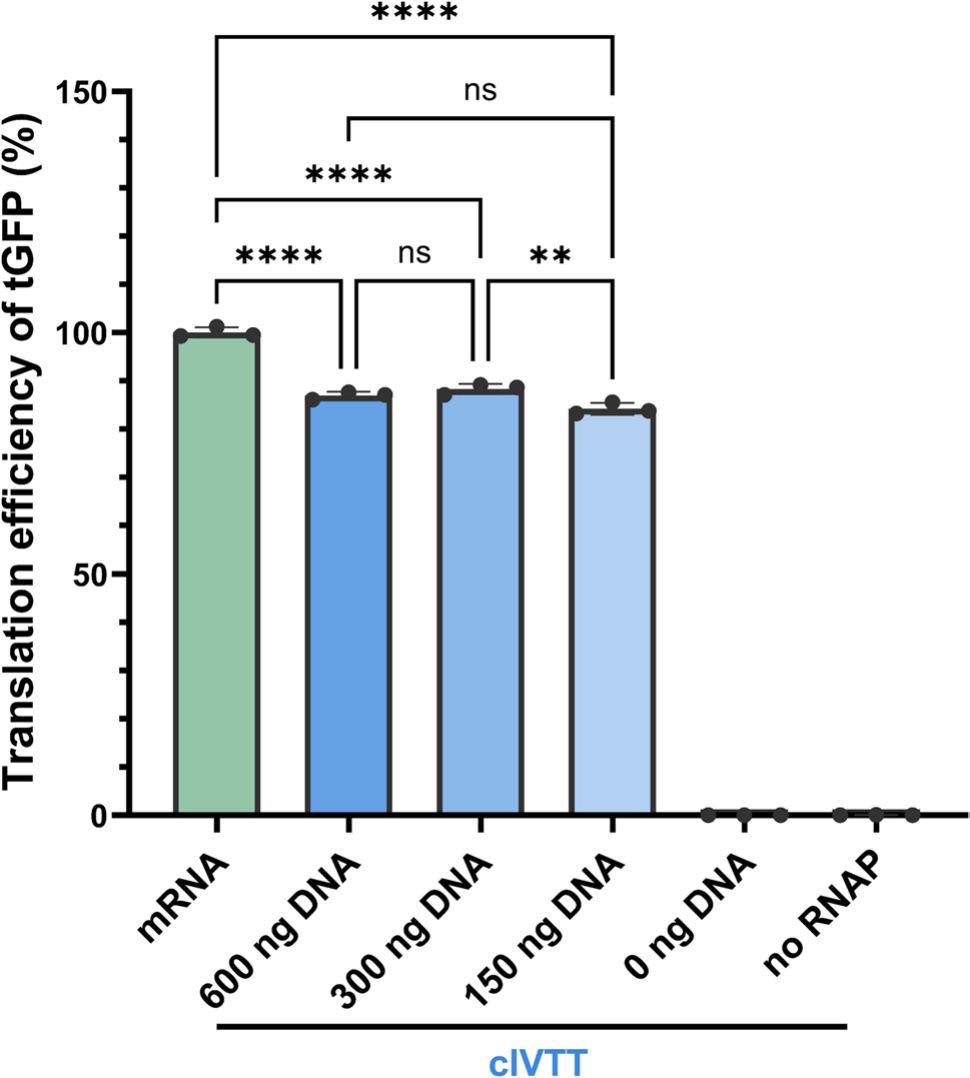
DNA template concentration-dependent productivity of bilayer cIVTT. Bilayer cIVTT efficiency at 20 °C was quantified by measuring the fluorescence intensity of synthesised tGFP using mRNA as template for the conventional two-step method and comparing it with a range of linearised DNA vector concentrations used as templates for cIVTT, as well as with reactions lacking either the translation template or T7 RNAP. Translation efficiency was calculated as the tGFP fluorescence in the cIVTT reaction under each condition divided by the tGFP fluorescence obtained in the two-step translation reaction using mRNA as template, and expressed as a percentage. Asterisks indicate statistically significant differences based on one-way ANOVA followed by Tukey’s post hoc test (**p < 0.01, ****p < 0.0001, *ns* = not significant at *α* = 0.05). Error bars represent mean ± standard deviation, *n* = 3 independent replicates per condition

The productivity of the cIVTT system was assessed by measuring the fluorescence of reaction mixtures containing increasing concentrations of a linearised, T7 promoter-driven pEU3-NII vector encoding tGFP, following overnight incubation. The results demonstrated that tGFP yields obtained using the coupled system were comparable to those achieved with the conventional bilayer two-step method, in which transcription and translation are performed in separate reactions (Fig. 1). Notably, cIVTT exhibited low sensitivity to DNA template concentration, as tGFP production showed minimal variation across template inputs ranging from 150 to 600 ng (Fig. 1).

**Fig. 2:**
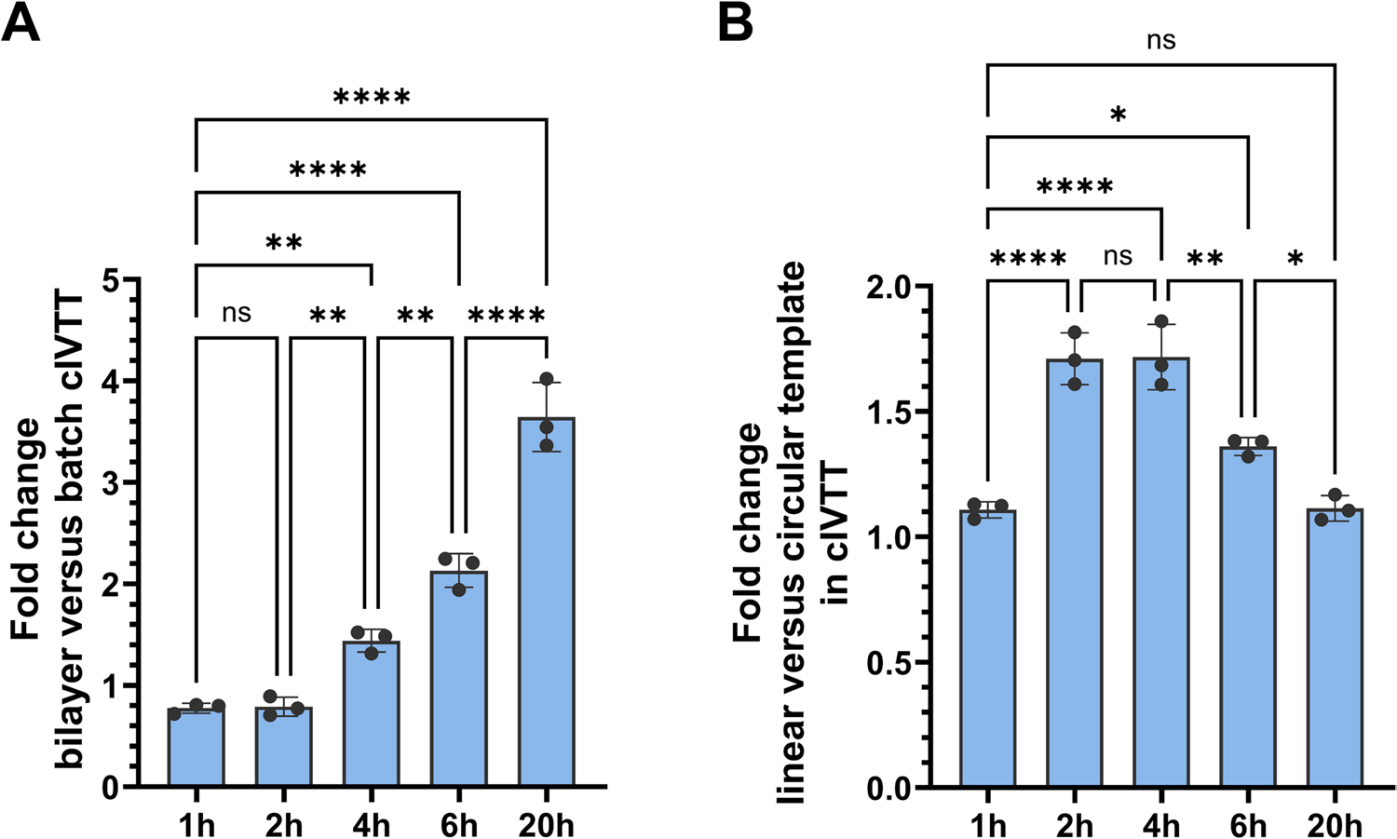
Superior productivity of bilayer vs. batch format and linearised vs. circular DNA templates in cIVTT. **A** Fold change of tGFP fluorescence intensity for bilayer versus batch cIVTT reactions using linearised pEU3-NII-tGFP as template after 1, 2, 4, 6, 20 h of incubation at 20 °C. Fold change was calculated as the fluorescence of tGFP in the bilayer reaction divided by the fluorescence of tGFP in the batch reaction at each timepoint, with the batch condition taken as reference. **B** Fold change of tGFP fluorescence intensity for reactions using linearised versus circular pEU3-NII-tGFP as template in bilayer cIVTT after 1, 2, 4, 6, 20 h of incubation. Fold change was calculated as the fluorescence of tGFP using linearised template divided by the fluorescence of tGFP using circular template at each timepoint, with the circular template taken as reference. **A**, **B** Asterisks indicate statistically significant differences based on one-way ANOVA followed by Tukey’s post hoc test (*p < 0.05, **p < 0.01, ****p < 0.0001, *ns* = not significant at *α* = 0.05). Error bars represent mean ± standard deviation, *n* = 3 independent replicates per timepoint.

Following demonstration of applicability of cIVTT using a T7 promoter-driven vector, its performance was quantitatively compared with batch‑format in vitro translation. Reaction mixtures containing linearised pEU3‑NII‑tGFP were prepared, and tGFP synthesis was monitored by fluorescence measurement at defined timepoints over a 20-h incubation period. During the initial phase of the reaction, no significant difference in protein yield was observed between the two formats (at 1- and 2-h incubation); however, from approximately 4 h onward, bilayer cIVTT reproducibly exhibited higher productivity than the batch format, reaching 3.64 ± 0.34-fold higher productivity at 20 h incubation (Fig. 2A).

Although the use of circular plasmid DNA obviates the need for vector linearisation and thereby simplifies the experimental workflow, conflicting reports exist regarding the influence of template topology on the performance of T7 RNAP-based in vitro transcription–translation systems (Zhao et al. 2007; Nagy et al. 2020). To experimentally address this contradiction, cIVTT reactions were performed using either linearised or circular pEU3‑NII‑tGFP templates. Linearised DNA consistently yielded higher tGFP fluorescence levels and demonstrated peak productivity advantage between 2 and 4 h (1.61 ± 0.13-fold at 4 h incubation). While the magnitude of this effect decreased after approximately 6 h of incubation, linearised templates retained a small advantage over circular DNA throughout the remainder of the reaction (fold change = 1.11 ± 0.05) (Fig. 2B).

### Determining the optimal reaction temperature

**Fig. 3:**
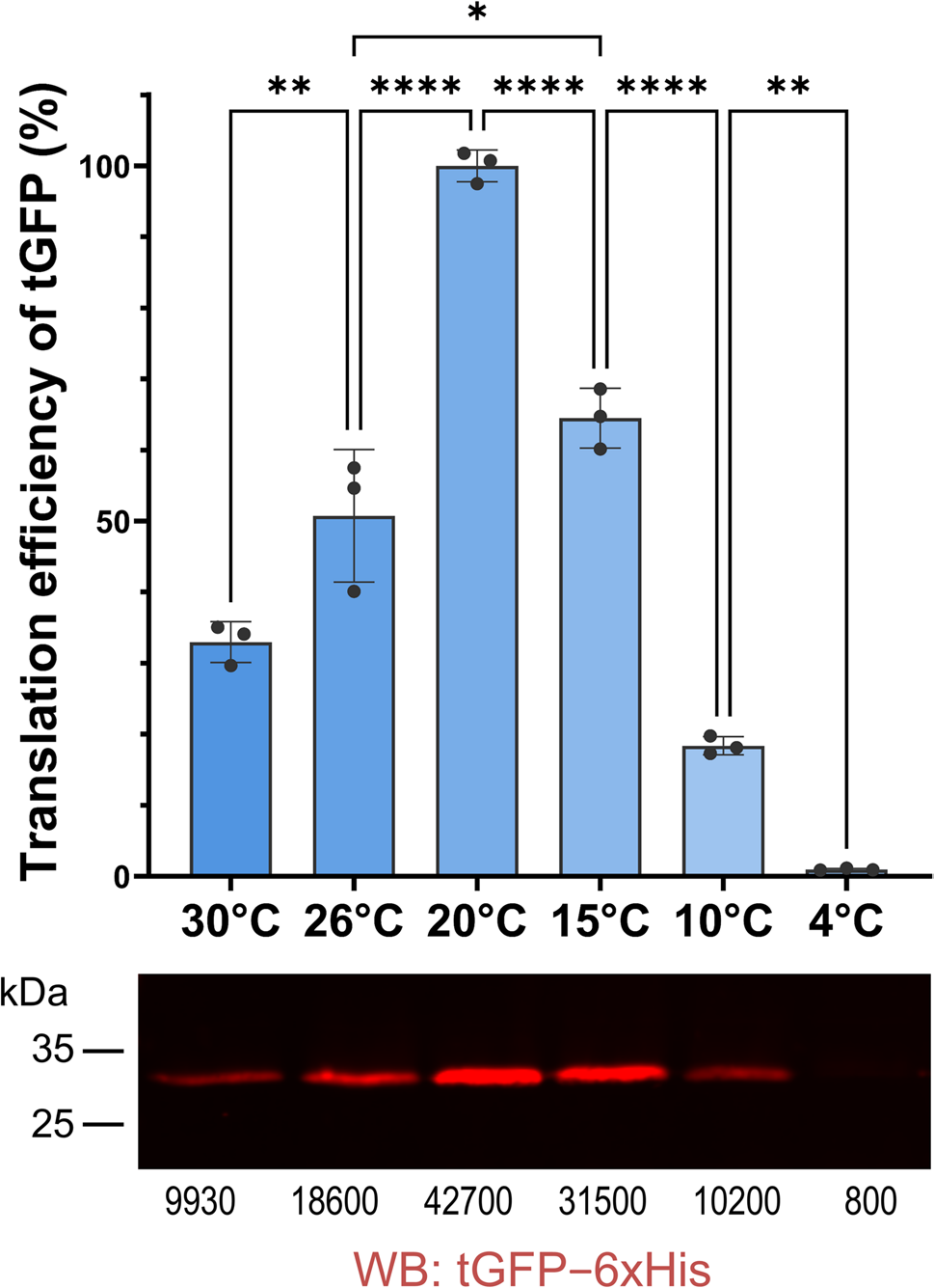
Temperature-dependent productivity of cIVTT. Temperature-dependent cIVTT efficiency was quantified by measuring the fluorescence intensity of synthesised tGFP in the crude wheat germ extract at various temperatures and normalising these values to the mean fluorescence intensity at 20 °C, and expressed as a percentage. One replicate from each temperature condition was analysed by western blotting with anti-polyHistidine antibodies, and the results are shown below the graph with the band intensities indicated in arbitrary units. Expected molecular weight of tGFP–6xHis is 26.8 kDa. Asterisks indicate statistically significant differences based on one-way ANOVA followed by Tukey’s post hoc test (*p < 0.05, **p < 0.01, ****p < 0.0001 at α = 0.05). Error bars represent mean ± standard deviation, *n* = 3 independent replicates per condition

Optimisation of reaction parameters, including temperature, across different in vitro translation systems is critical for maximising protein yield and ensuring experimental reproducibility. Bacteriophage RNA polymerases exhibit maximal transcriptional activity around 37 °C, matching the optimal growth temperature of *Escherichia coli*, whereas protein synthesis in WGE-based systems is favoured at lower temperatures (15–25 °C). At higher temperatures, translational activity of the extract is reduced, and proper protein folding may be compromised, collectively resulting in decreased soluble protein yield. To determine the optimal temperature for cIVTT using wheat germ extract, tGFP expression reactions were performed at six defined temperatures. Fluorescence measurements after overnight incubation revealed a clear relationship between temperature and cIVTT productivity. Negligible signal was observed at 4 °C, and the yield increased up to 20 °C, but declined at 26 °C. Notably, lower temperatures appear to provide a better compromise between the differing optimal conditions of T7 RNA polymerase and the wheat translation machinery, as tGFP translation efficiency at 15 °C exceeded that at 26 °C (64.49% vs. 50.75%) (Fig. 3).

Next, we determined the tGFP yield at 20 °C using a fluorescence calibration curve generated from purified tGFP–6xHis expressed in *E. coli* and spiked into crude wheat germ extract. We estimated a concentration of 79.81 ng/µL of tGFP–6xHis in the 56.5 µL small-scale reaction containing 2.5 µL WGE, corresponding to 1804 µg target protein per mL WGE (Supplementary Table S1).

### Assessing general applicability of cIVTT

To demonstrate the broad applicability of the optimised cIVTT platform, expression of two human and one plant protein was compared between the conventional two‑step bilayer system and cIVTT by western blot analysis. Linearised expression vectors encoding GST‑tagged human cadherin‑11 extracellular domains EC1–EC3 (CDH11) and *Arabidopsis thaliana* RUB1‑conjugating enzyme 2 (RCE2) were used as templates either for two-step in vitro transcription followed by translation or directly in cIVTT reactions.

**Fig. 4:**
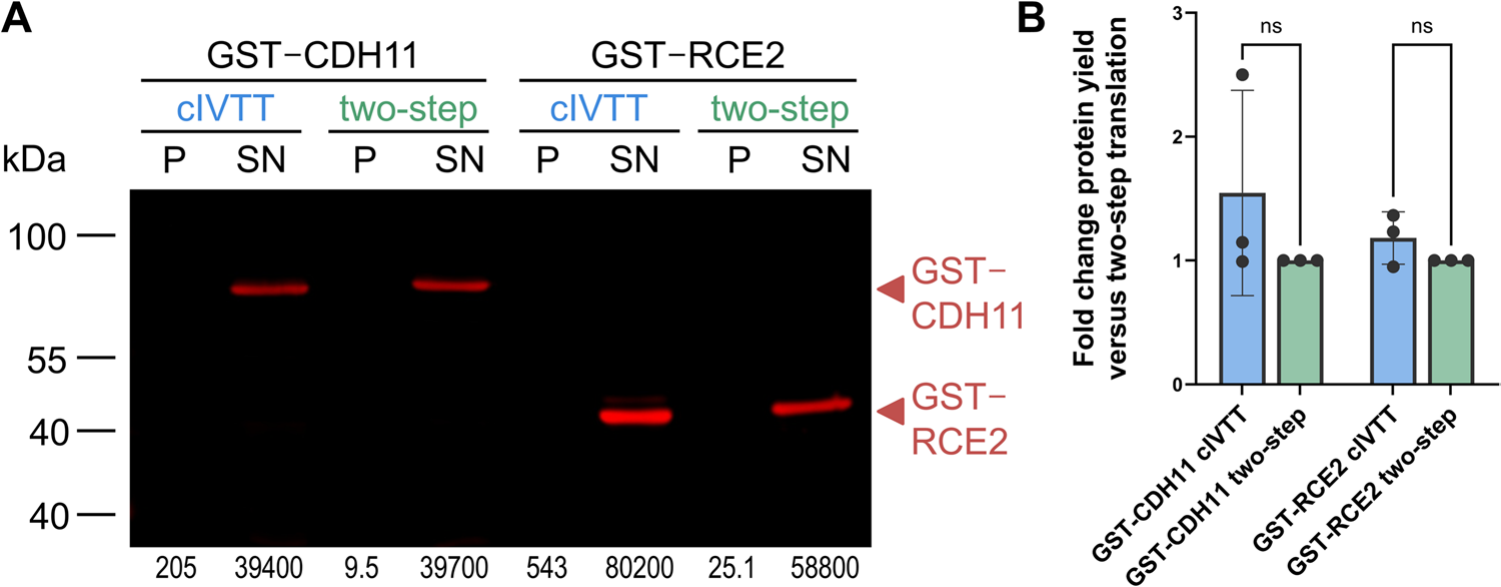
Comparison of coupled and two‑step in vitro translation of GST–CDH11 and GST–RCE2 fusion proteins. Proteins were expressed using either a cIVTT or two‑step bilayer strategy. For each method, both the pellet (P) and supernatant (SN) fractions were analysed. **A** Samples were separated by SDS–PAGE and detected by western blot using GST-specific primary and near-infrared fluorescently labelled secondary antibodies. Molecular weight markers (left) are indicated in kDa. The band intensities of the representative image are indicated below in arbitrary units. **B** Protein yields were quantified by densitometric analysis of band intensities and compared to those obtained with the two-step method for each independent experiment. Statistical significance was determined using one-way ANOVA followed by Tukey’s post hoc test (*ns* = not significant at *α* = 0.05). Error bars represent mean ± standard deviation (*n* = 3 independent replicates). Expected molecular weights of expressed proteins: GST–CDH11 63.1 kDa, GST–RCE2 47.1 kDa

After overnight incubation at 20 °C and prior to SDS–PAGE, reaction mixtures were centrifuged and soluble (supernatant) and insoluble (pellet) fractions were analysed separately. Western blot detection using a GST‑specific antibody revealed that both CDH11 and RCE2 were predominantly present in the soluble fraction (Fig. 4A). Importantly, comparable protein yields were obtained with both the conventional two-step and cIVTT bilayer formats, indicating that coupling transcription and translation does not compromise expression efficiency or protein solubility of the studied proteins in the WGE-based in vitro translation system (Fig. 4B).

**Fig. 5:**
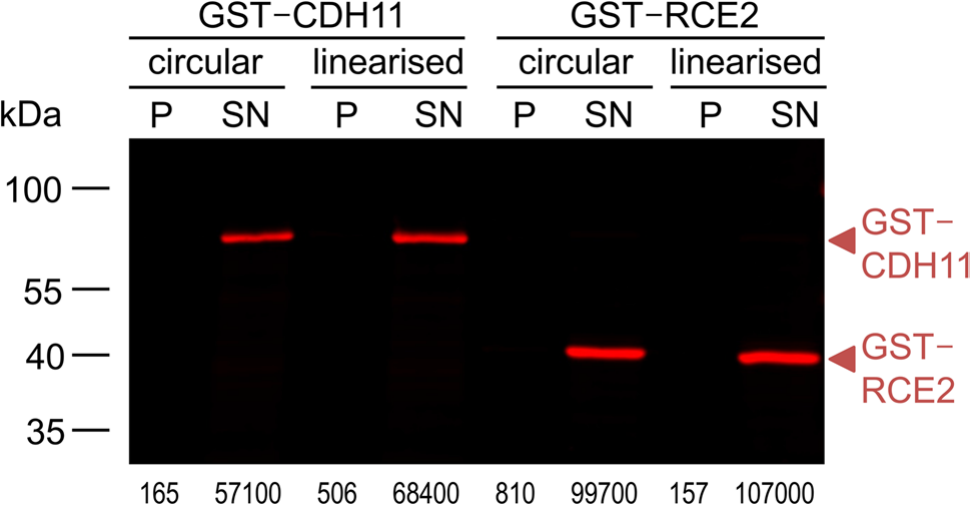
Effect of vector topology on GST–CDH11 and GST–RCE2 protein expression using cIVTT. Proteins were expressed by cIVTT using either linearised or circular vectors as reaction templates. For each condition, both the pellet (P) and supernatant (SN) fractions were analysed. Samples were separated by SDS–PAGE and detected by western blot using GST-specific primary and near-infrared fluorescently labelled secondary antibodies. Molecular weight markers (left) are indicated in kDa. The band intensities are indicated below in arbitrary units. Expected molecular weights of expressed proteins: GST–CDH11 63.1 kDa, GST–RCE2 47.1 kDa

Fluorescence measurements from cIVTT reactions producing tGFP with either linearised or circular plasmid templates showed that the initial advantage of the linearised template diminished over time and became minimal after overnight incubation. To assess whether this observation is general, GST–CDH11 and GST–RCE2 were expressed under the same conditions from linearised and circular vectors, and the reaction mixtures were fractionated into soluble and insoluble protein pools. Western blotting with an anti‑GST antibody revealed comparable signals for both template formats in all fractions, indicating there are negligible differences in productivity between linear and circular templates (Fig. 5).

Production of human cardiac troponin I (cTnI) is considered technically challenging, as recombinant expression in prokaryotic systems requires codon optimisation and protein purification under denaturing conditions (Al‐Hillawi et al. 1994). To evaluate whether cell-free in vitro translation in cIVTT format is suitable to produce this difficult-to-express protein, both the conventional and the cIVTT bilayer reaction formats were assembled using an N-terminally GST-tagged cTnI construct encoded by the pEU3-NII-GLICNot vector, without codon optimisation of cTnI. Reactions were performed at two different temperatures, i.e. at 20 °C and 15 °C, which had produced the highest and second-highest tGFP yields in our experiments (Fig. 3).

**Fig. 6:**
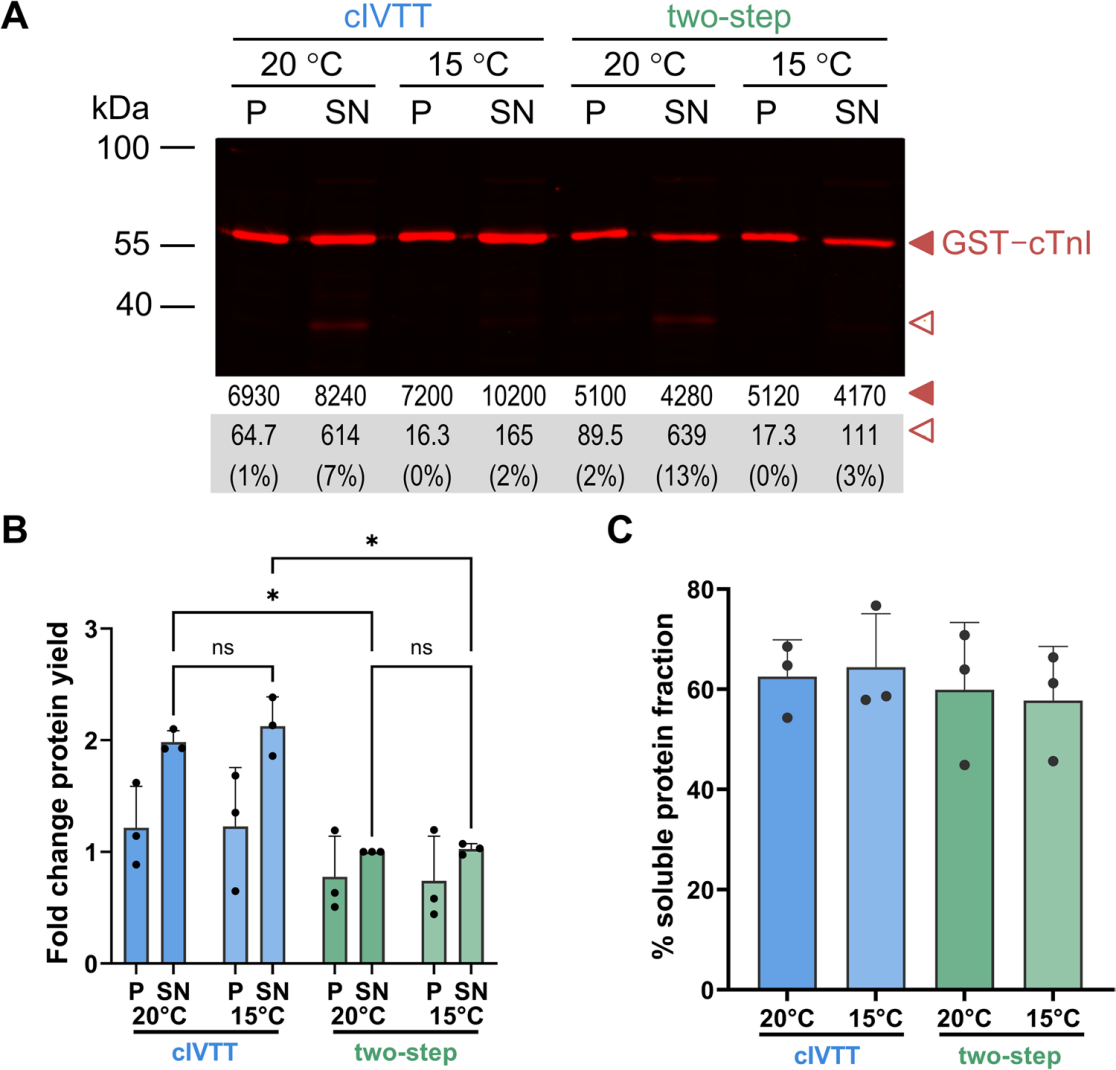
Comparison of coupled and two‑step in vitro translation of GST–cTnI fusion protein at various temperatures. The human cTnI protein was expressed using either a cIVTT or two‑step bilayer strategy at 20 °C or 15 °C. For each strategy, both the pellet (P) and supernatant (SN) fractions were analysed. **A** Representative image of samples separated by SDS–PAGE and detected by western blot using GST-specific primary and near-infrared fluorescently labelled secondary antibodies. GST–cTnI is marked with a filled triangle, and the additional anti-GST-detected band is marked with an open triangle. Molecular weight markers (left) are indicated in kDa. The band intensities of GST–cTnI and the additional band in the representative image are indicated below in arbitrary units. The additional band was quantified as the ratio of its intensity to the sum of both band intensities, expressed as a percentage. The expected molecular weight of GST–cTnI is 52.8 kDa. **B** Fold changes in protein yield versus soluble fraction of two-step translation at 20 °C, after 20 h of incubation. The values were calculated as the cTnI band intensity divided by the band intensity of soluble cTnI in the two-step reaction at 20 °C for each independent experiment. **C** Percentage of soluble protein fraction for coupled and two-step translation at 15 °C and 20 °C. The values were calculated as the soluble cTnI band intensity divided by the sum of soluble and precipitate cTnI band intensities at a given temperature and method for each independent experiment, and expressed as a percentage. **B**, **C** Band intensities were quantified by densitometry and analysed using two-way ANOVA with Tukey’s post hoc test (*p < 0.05). Error bars represent mean ± standard deviation (*n* = 3 independent replicates per each condition)

Following overnight incubation, reaction mixtures were separated into soluble and insoluble fractions by centrifugation. Supernatant and pellet fractions were analysed by SDS–PAGE and western blotting using a GST-specific antibody (Fig. 6A). The fluorescence signal measurements showed that the overall cTnI yield was approximately twofold higher in the cIVTT setup at both temperatures. Although productivity was unaffected by decreasing temperature in either setup (Fig. 6B), no substantial difference in protein solubility was observed either: approximately 60% of the produced cTnI partitioned into the soluble fraction in both systems at both temperatures (Fig. 6C). Of note, an additional lower molecular weight faint band was detected with anti-GST antibodies in the soluble fractions predominantly at 20 °C and to a lesser extent at 15 °C (Fig. 6A). At 20 °C, this band represented ~ 7% and ~ 13% of the GST-tagged proteins with the cIVTT and two-step methods, respectively, whereas at 15 °C it accounted for only 2–3% with both methods.

## Discussion

The protocol presented here uses a modified pEU3‑NII vector driven by a T7 promoter, which possesses the 5′ m⁷G cap replacing GAA triplet and the native tobacco mosaic virus (TMV) 5’-leader sequence (called Ω) translation enhancer (Nagy et al. 2020). When applied in a bilayer cIVTT format using WGE, this vector yielded protein amounts comparable to those obtained with the conventional two‑step bilayer system (Supplementary Table S1). Considering the availability of protocols to produce highly efficient WGE in-house (Takai et al. 2010; Fogeron et al. 2017), these developments established a generally applicable WGE-based cIVTT as a practical and streamlined alternative for routine eukaryotic cell‑free protein synthesis. This approach thus offers an effective compromise—delivering higher productivity than conventional batch reactions and similar yields to two-step reactions, while remaining substantially simpler and more accessible than dialysis/CECF (continuous-exchange cell-free) formats—thereby providing a practical and scalable option for routine protein synthesis.

In order to further streamline the process, we compared the productivity of circular and linearised vectors in bilayer cIVTT. Our results showed that the linear template offered only a minor advantage in terms of protein yield, questioning the value of linearisation under high-throughput cIVTT conditions, as circular templates perform comparably well. The relatively high translation efficiency of circular templates when using T7 RNAP can be explained by the negative supercoil that is characteristic of plasmid preparations, which has been shown to facilitate promoter melting, enabling transcription initiation (Ma and Wang 2016). However, it should be noted that the ratio of supercoiled plasmid must be ensured by following an appropriate plasmid purification protocol to leverage this advantage of circular plasmids (Carbone et al. 2012). Furthermore, in cIVTT, the DNA template remains in the WGE for extended periods, exposing it to endogenous nucleases in the crude protein extract (Fogeron et al. 2021). Circular plasmids offer a significant advantage under these nuclease-rich conditions due to the absence of free DNA ends, which makes them more resistant to exonucleases, preserves template availability thus sustains mRNA polymerisation.

Due to the reduced catalytic rates of plant ribosomes at suboptimal temperatures, translation efficiency of WGE is expected to decline below 25 °C. Interestingly, our temperature‑optimisation experiments showed that the highest yield of fluorescent tGFP occurred at 20 °C, even though both T7 RNAP activity and ribosomal throughput are reduced at this temperature. It has been documented that lower temperatures enhance the solubility of recombinant proteins in bacterial cells (Schein and Noteborn 1988). In an analogous manner, reducing the reaction temperature to 20 °C in WGE likely slows translation just enough to promote more efficient co‑translational folding, limiting aggregation of partially folded intermediates, and stabilising the β‑barrel during GFP chromophore maturation (Pédelacq et al. 2005). As a result, a greater fraction of GFP attains its fully folded, fluorescent conformation, compensating for—and ultimately outweighing—the modest decrease in overall translation rate. Our cTnI expression data indicate that further lowering the incubation temperature did not compromise productivity, yet it also did not improve solubility. However, at 15 °C, we observed a significantly reduced amount of the additional lower molecular weight anti-GST-detected band in the GST–cTnI-containing extract, which may have arisen from premature termination of translation or the activity of an endogenous wheat germ extract protease (Okimune et al. 2020). Overall, reduced temperatures can be a useful lever to increase the soluble fraction of properly folded appropriate length protein and therefore warrant empirical testing on a case‑by‑case basis.

Our studies were limited to the production of cytosolic eukaryotic proteins; therefore, we cannot provide experimental data on the synthesis of disulphide bond-containing or membrane proteins. Nevertheless, the presented cIVTT system is expected to perform under these conditions, provided that the supplements applied for these applications do not impair transcription or translation. In DTT-free bilayer arrangements developed for the production of disulphide bond-containing proteins, the lower layer—where the majority of transcription and translation occurs—could support T7 RNA polymerase activity (Buntru et al. 2015). Furthermore, it has been demonstrated that T7 RNA polymerase remains active in liposome-based artificial cell systems (Monnard et al. 2007; Takahashi and Ogawa 2021). Notwithstanding, these hypotheses require experimental validation.

In summary, the presented results demonstrate that WGE-based bilayer cIVTT can be made highly effective when its inherent constraints are addressed through thoughtful vector design, template topology, and temperature optimisation. By integrating a T7‑based expression cassette, employing circular plasmid templates that resist exonuclease degradation, and selecting a reaction temperature that balances translational efficiency with proper protein folding, the cIVTT system achieved protein yields that are comparable to those of the conventional two‑step WGE–based cell-free protein synthesis workflow. Collectively, these developments established WGE-cIVTT as a practical and streamlined alternative to two-step reactions at microscale for routine eukaryotic cell‑free protein synthesis.

## Supplementary Information

Below is the link to the electronic supplementary material.

Supplementary Material 1 (PDF 427 KB)

## Data Availability

The data presented in the study will be made available by the corresponding author upon request.

## Abbreviations

CDH11: Human Cadherin-11
CFPS: Cell-free protein synthesis
cIVTT: Coupled in vitro transcription–translation
cTnI: Human cardiac muscle Troponin I
GFP: Green fluorescent protein
GST: Glutathione S-transferase
NTP: Nucleoside triphosphates
PBS: Phosphate buffer saline
RCE2: *Arabidopsis thaliana* RUB1-conjugating enzyme 2
RNAP: RNA polymerase
WGE: Wheat germ extract
