# Supplementary material for: Streamlined cell-free protein synthesis using wheat germ extract
Source: Appl Microbiol Biotechnol. 2026 Jun 11;110:253. doi: 10.1007/s00253-026-13914-x (PMC13481492; doi:10.1007/s00253-026-13914-x)
Supplement: Supplementary file 1 [file 253_2026_13914_MOESM1_ESM.pdf]

Supplementary material for

Streamlined cell-free protein synthesis using wheat germ extract

Zsuzsanna Szeitner[1], Alexandra Krisztina Tar[1], Ákos Harkai[1], Anna Gyurkovics[1], Tamás Mészáros[1], and Brigitta Margit Kállai[1,*]

[1]Department of Molecular Biology, Institute of Biochemistry and Molecular Biology, Semmelweis University, 1094 Budapest, Tűzoltó u. 37–47

*Correspondence: kallai.brigitta@semmelweis.hu

Contents

Supplementary Table S1. Expected yields of target proteins obtained using wheat germ extract with different methods

Supplementary Fig. S1. Coding DNA sequences of the expressed proteins

Supplementary Fig. S2. Fluorescence spectra of expressed tGFP

Supplementary Fig. S3. Fig. 3 uncropped western blot with densitometry

Supplementary Fig. S4. Fig. 4 uncropped western blot with densitometry

Supplementary Fig. S5. Fig. 5 uncropped western blot with densitometry

Supplementary Fig. S6. Fig. 6 uncropped western blot with densitometry

Supplementary Table S1. Expected yields of target proteins obtained using wheat germ extract with different methods

Method	Setup	Template	RNAP	Wheat germ extract	Incubation temp.	Incubation time (hours)	Yield (µg protein /1 mL of WGE)[1]	Quantified protein	Purified	Reference
Coupled	Bilayer	linear vector	T7	WEPRO7240 (CellFree Sciences)	30°C	20	591	tGFP	no	This paper
Coupled	Bilayer	linear vector	T7	WEPRO7240 (CellFree Sciences)	26°C	20	913	tGFP	no	This paper
Coupled	Bilayer	linear vector	T7	WEPRO7240 (CellFree Sciences)	20°C	20	1804	tGFP	no	This paper
Coupled	Bilayer	linear vector	T7	WEPRO7240 (CellFree Sciences)	15°C	20	1161	tGFP	no	This paper
Coupled	Bilayer	circular vector	T7	WEPRO7240 (CellFree Sciences)	20°C	20	1904	tGFP	no	This paper
Coupled	Bilayer	circular vector	T7	WEPRO7240 (CellFree Sciences)	15°C	20	1475	tGFP	no	This paper
Coupled	Bilayer	linear vector PCR-generated DNA	SP6	WEPRO TT (CellFree Sciences)	26°C	24	400	N/A	N/A	Premium ONE Expression Kit (CellFree Sciences)
Coupled	Batch	PCR-generated DNA	T7	WEPRO1240 (CellFree Sciences)	30°C	3	425	YPet	no	Takahashi 2021
Coupled	Batch	PCR-generated DNA	SP6	TnT® SP6 High-Yield Wheat Germ Master Mix (Promega)	30°C	3	33,3	YPet	no	Takahashi 2021
Coupled	Batch	circular vector	SP6	TnT® SP6 High-Yield Wheat Germ Master Mix (Promega)	25°C	2	130–300	various	no	Zhao 2007
Coupled	Batch	circular vector PCR-generated DNA	SP6	TnT® SP6 High-Yield Wheat Germ Master Mix (Promega)	30°C	3	16,6–166	N/A	N/A	TnT® SP6 High-Yield Wheat Germ Protein Expression System (Promega)
Two-step	Bilayer	linear vector PCR-generated DNA	SP6	WEPRO1240 (CellFree Sciences)	15°C	20	441	GFP	yes	WEPRO1240 Expression Kit (CellFree Sciences)
Two-step	Bilayer	linear vector PCR-generated DNA	SP6	WEPRO7240 (CellFree Sciences)	15°C	20	849	GFP	yes	WEPRO7240 Core Kit (CellFree Sciences)
Two-step	Bilayer	linear vector PCR-generated DNA	SP6	WEPRO9240 (CellFree Sciences)	15°C	20	2300	CDC27	no	FLEXIQuant PLUS Expression Kit (CellFree Sciences)
Two-step	Bilayer	mRNA	SP6	Home-made	22°C	16	200–1000	various	yes	Fogeron 2017, Fogeron 2021
Two-step	Bilayer	mRNA	-	not available	15–26°C	up to 24	3200	GFP	no	Harbers 2014
Two-step	Batch	mRNA	-	not available	15–26°C	few	1600	GFP	no	Harbers 2014

[1]In case of TnT® SP6 High-Yield Wheat Germ Master Mix a transcription-translation mixture provided by the manufacturer in which the amount of WGE is not disclosed, we assumed that the Master Mix volume is equal to WGE volume.

References

Fogeron ML, Badillo A, Penin F, Böckmann A (2017) Wheat Germ Cell-Free Overexpression for the Production of Membrane Proteins. Methods in Molecular Biology 1635:91–108. https://doi.org/10.1007/978-1-4939-7151-0_5

Fogeron ML, Lecoq L, Cole L, Harbers M, Böckmann A (2021) Easy Synthesis of Complex Biomolecular Assemblies: Wheat Germ Cell-Free Protein Expression in Structural Biology. Front Mol Biosci 8:639587. https://doi.org/10.3389/fmolb.2021.639587

Harbers, M. (2014). Wheat germ systems for cell-free protein expression. FEBS letters, 588(17), 2762-2773. https://doi.org/10.1016/j.febslet.2014.05.061

Takahashi H, Ogawa A (2021) Coupled in vitro transcription/translation based on wheat germ extract for efficient expression from PCR-generated templates in short-time batch reactions. Bioorg Med Chem Lett 52:128412. https://doi.org/10.1016/j.bmcl.2021.128412

Zhao KQ, Hurst R, Slater MR, Bulleit RF (2007) Functional protein expression from a DNA based wheat germ cell-free system. Journal of Structural and Functional Genomics 2007 8:4 8:199–208. https://doi.org/10.1007/s10969-007-9035-2

Supplementary Fig. S1. Coding DNA sequences of the expressed proteins

Green: 6xHis tag
Red: GST tag
Black: linker sequence including TEV recognition site
Blue: target protein coding sequence

pEU3-NII-tGFP

ATGGAGAGCGACGAGAGCGGCCTGCCCGCCATGGAGATCGAGTGCCGCATCACCGGCACCCTGAACGGCGTGGAGTTCGAGCTGGTGGGCGGCGGAGAGGGC
ACCCCCGAGCAGGGCCGCATGACCAACAAGATGAAGAGCACCAAAGGCGCCCTGACCTTCAGCCCCTACCTGCTGAGCCACGTGATGGGCTACGGCTTCTAC
CACTTCGGCACCTACCCCAGCGGCTACGAGAACCCCTTCCTGCACGCCATCAACAACGGCGGCTACACCAACACCCGCATCGAGAAGTACGAGGACGGCGGC
GTGCTGCACGTGAGCTTCAGCTACCGCTACGAGGCCGGCCGCGTGATCGGCGACTTCAAGGTGATGGGCACCGGCTTCCCCGAGGACAGCGTGATCTTCACC
GACAAGATCATCCGCAGCAACGCCACCGTGGAGCACCTGCACCCCATGGGCGATAACGATCTGGATGGCAGCTTCACCCGCACCTTCAGCCTGCGCGACGGC
GGCTACTACAGCTCCGTGGTGGACAGCCACATGCACTTCAAGAGCGCCATCCACCCCAGCATCCTGCAGAACGGGGGCCCCATGTTCGCCTTCCGCCGCGTG
GAGGAGGATCACAGCAACACCGAGCTGGGCATCGTGGAGTACCAGCACGCCTTCAAGACCCCGGATGCAGATGCCGGTGAAGAACTCGAGCACCACCACCAC
CACCACTGA

pEU3-NII-GLICNot_CDH11 (extracellular domains: EC1, EC2, EC3)

ATGTCCCCTATACTAGGTTATTGGAAAATTAAGGGCCTTGTGCAACCCACTCGACTTCTTTTGGAATATCTTGAAGAAAAATATGAAGAGCATTTGTATGAG
CGCGATGAAGGTGATAAATGGCGAAACAAAAAGTTTGAATTGGGTTTGGAGTTTCCCAATCTTCCTTATTATATTGATGGTGATGTTAAATTAACACAGTCT
ATGGCCATCATACGTTATATAGCTGACAAGCACAACATGTTGGGTGGTTGTCCAAAAGAGCGTGCAGAGATTTCAATGCTTGAAGGAGCGGTTTTGGATATT
AGATACGGTGTTTCGAGAATTGCATATAGTAAAGACTTTGAAACTCTCAAAGTTGATTTTCTTAGCAAGCTACCTGAAATGCTGAAAATGTTCGAAGATCGT
TTATGTCATAAAACATATTTAAATGGTGATCATGTAACCCATCCTGACTTCATGTTGTATGACGCTCTTGATGTTGTTTTATACATGGACCCAATGTGCCTG
GATGCGTTCCCAAAATTAGTTTGTTTTAAAAAACGTATTGAAGCTATCCCACAAATTGATAAGTACTTGAAATCCAGCAAGTATATAGCATGGCCTTTGCAG
GGCTGGCAAGCCACGTTTGGTGGTGGCGACCATCCTCCAAAATCGGATGAGAACCTGTACTTCCAATCCAATGCAGGCTGGGTCTGGAACCAGTTCTTCGTG
ATAGAGGAGTACACCGGGCCTGACCCCGTGCTTGTGGGCAGGCTTCATTCAGATATTGACTCTGGTGATGGGAACATTAAATACATTCTCTCAGGGGAAGGA
GCTGGAACCATTTTTGTGATTGATGACAAATCAGGGAACATTCATGCCACCAAGACGTTGGATCGAGAAGAGAGAGCCCAGTACACGTTGATGGCTCAGGCG
GTGGACAGGGACACCAATCGGCCACTGGAGCCACCGTCGGAATTCATTGTCAAGGTCCAGGACATTAATGACAACCCTCCGGAGTTCCTGCACGAGACCTAT
CATGCCAACGTGCCTGAGAGGTCCAATGTGGGAACGTCAGTAATCCAGGTGACAGCTTCAGATGCAGATGACCCCACTTATGGAAATAGCGCCAAGTTAGTG
TACAGTATCCTCGAAGGACAACCCTATTTTTCGGTGGAAGCACAGACAGGTATCATCAGAACAGCCCTACCCAACATGGACAGGGAGGCCAAGGAGGAGTAC
CACGTGGTGATCCAGGCCAAGGACATGGGTGGACATATGGGCGGACTCTCAGGGACAACCAAAGTGACGATCACACTGACCGATGTCAATGACAACCCACCA
AAGTTTCCGCAGAGCGTATACCAGATGTCTGTGTCAGAAGCAGCCGTCCCTGGGGAGGAAGTAGGAAGAGTGAAAGCTAAAGATCCAGACATTGGAGAAAAT
GGCTTAGTCACATACAATATTGTTGATGGAGATGGTATGGAATCGTTTGAAATCACAACGGACTATGAAACACAGGAGGGGGTGATAAAGCTGAAAAAGCCT
GTAGATTTTGAAACCAAAAGAGCCTATAGCTTGAAGGTAGAGGCAGCCAACGTGCACATCGACCCGAAGTTTATCAGCAATGGCCCTTTCAAGGACACTGTG
ACCGTCAAGATCTCAGTAGAAGATGCTGATGAGCCCCCTATGTTCTGA

pEU3-NII-GLICNot_RCE2

ATGTCCCCTATACTAGGTTATTGGAAAATTAAGGGCCTTGTGCAACCCACTCGACTTCTTTTGGAATATCTTGAAGAAAAATATGAAGAGCATTTGTATGAG
CGCGATGAAGGTGATAAATGGCGAAACAAAAAGTTTGAATTGGGTTTGGAGTTTCCCAATCTTCCTTATTATATTGATGGTGATGTTAAATTAACACAGTCT
ATGGCCATCATACGTTATATAGCTGACAAGCACAACATGTTGGGTGGTTGTCCAAAAGAGCGTGCAGAGATTTCAATGCTTGAAGGAGCGGTTTTGGATATT
AGATACGGTGTTTCGAGAATTGCATATAGTAAAGACTTTGAAACTCTCAAAGTTGATTTTCTTAGCAAGCTACCTGAAATGCTGAAAATGTTCGAAGATCGT
TTATGTCATAAAACATATTTAAATGGTGATCATGTAACCCATCCTGACTTCATGTTGTATGACGCTCTTGATGTTGTTTTATACATGGACCCAATGTGCCTG
GATGCGTTCCCAAAATTAGTTTGTTTTAAAAAACGTATTGAAGCTATCCCACAAATTGATAAGTACTTGAAATCCAGCAAGTATATAGCATGGCCTTTGCAG
GGCTGGCAAGCCACGTTTGGTGGTGGCGACCATCCTCCAAAATCGGATGAGAACCTGTACTTCCAATCCAATGCAATGATTGGGTTATTCAAAGTGAAAGAG
AAGCAAAGAGAAGAGTCTCAAAGTAACAACGGAAGAGGAGCTTCTACTGTCAAGAAGCAATCTGCTGGTGAACTTCGTCTTCACAAAGATATATCGGAGCTG
AACTTACCAAAGTCTTGTAAGATATCTTTTCCGAATGGGAAGAATGACTTGATGAATTTTGAAGTAACCATTAAACCTGATGAAGGATATTACTTGAGTGGT
AACTTTGTGTTCTCATTCCAAGTCTCTAATATGTACCCCCACGAAGCTCCTAAAGTGAAATGCAAGACCAAGGTGTATCATCCGAATATCGATCTAGAAGGA
AACGTCTGTCTGAACATCTTGCGTGAAGACTGGAAACCGGTTCTCAACATAAACACAGTTATCTACGGTCTTTTCCATCTCTTCACGGAACCCAATTACGAG
GATCCTCTGAACCATGAAGCAGCTGCAGTGTTGAGGGATAACCCGAAGACGTTCGAGTATAATGTCAGAAGGGCAATGATGGGTGGCCAAGTTGGCCAAACC
TCATTTCCTCGCTGCATGTGA

pEU3-NII-GLICNot_TNNI3 (cTnI)

ATGTCCCCTATACTAGGTTATTGGAAAATTAAGGGCCTTGTGCAACCCACTCGACTTCTTTTGGAATATCTTGAAGAAAAATATGAAGAGCATTTGTATGAG
CGCGATGAAGGTGATAAATGGCGAAACAAAAAGTTTGAATTGGGTTTGGAGTTTCCCAATCTTCCTTATTATATTGATGGTGATGTTAAATTAACACAGTCT
ATGGCCATCATACGTTATATAGCTGACAAGCACAACATGTTGGGTGGTTGTCCAAAAGAGCGTGCAGAGATTTCAATGCTTGAAGGAGCGGTTTTGGATATT
AGATACGGTGTTTCGAGAATTGCATATAGTAAAGACTTTGAAACTCTCAAAGTTGATTTTCTTAGCAAGCTACCTGAAATGCTGAAAATGTTCGAAGATCGT
TTATGTCATAAAACATATTTAAATGGTGATCATGTAACCCATCCTGACTTCATGTTGTATGACGCTCTTGATGTTGTTTTATACATGGACCCAATGTGCCTG
GATGCGTTCCCAAAATTAGTTTGTTTTAAAAAACGTATTGAAGCTATCCCACAAATTGATAAGTACTTGAAATCCAGCAAGTATATAGCATGGCCTTTGCAG
GGCTGGCAAGCCACGTTTGGTGGTGGCGACCATCCTCCAAAATCGGATGAGAACCTGTACTTCCAATCCAATGCAATGGCGGATGGGAGCAGCGATGCGGCT
AGGGAACCTCGCCCTGCACCAGCCCCAATCAGACGCCGCTCCTCCAACTACCGCGCTTATGCCACGGAGCCGCACGCCAAGAAAAAATCTAAGATCTCCGCC
TCGAGAAAATTGCAGCTGAAGACTCTGCTGCTGCAGATTGCAAAGCAAGAGCTGGAGCGAGAGGCGGAGGAGCGGCGCGGAGAGAAGGGGCGCGCTCTGAGC
ACCCGCTGCCAGCCGCTGGAGTTGGCCGGGCTGGGCTTCGCGGAGCTGCAGGACTTGTGCCGACAGCTCCACGCCCGTGTGGACAAGGTGGATGAAGAGAGA
TACGACATAGAGGCAAAAGTCACCAAGAACATCACGGAGATTGCAGATCTGACTCAGAAGATCTTTGACCTTCGAGGCAAGTTTAAGCGGCCCACCCTGCGG
AGAGTGAGGATCTCTGCAGATGCCATGATGCAGGCGCTGCTGGGGGCCCGGGCTAAGGAGTCCCTGGACCTGCGGGCCCACCTCAAGCAGGTGAAGAAGGAG
GACACCGAGAAGGAAAACCGGGAGGTGGGAGACTGGCGCAAGAACATCGATGCACTGAGTGGAATGGAGGGCCGCAAGAAAAAGTTTGAGAGCTGA

Supplementary Fig. S2. Fluorescence spectra of expressed tGFP

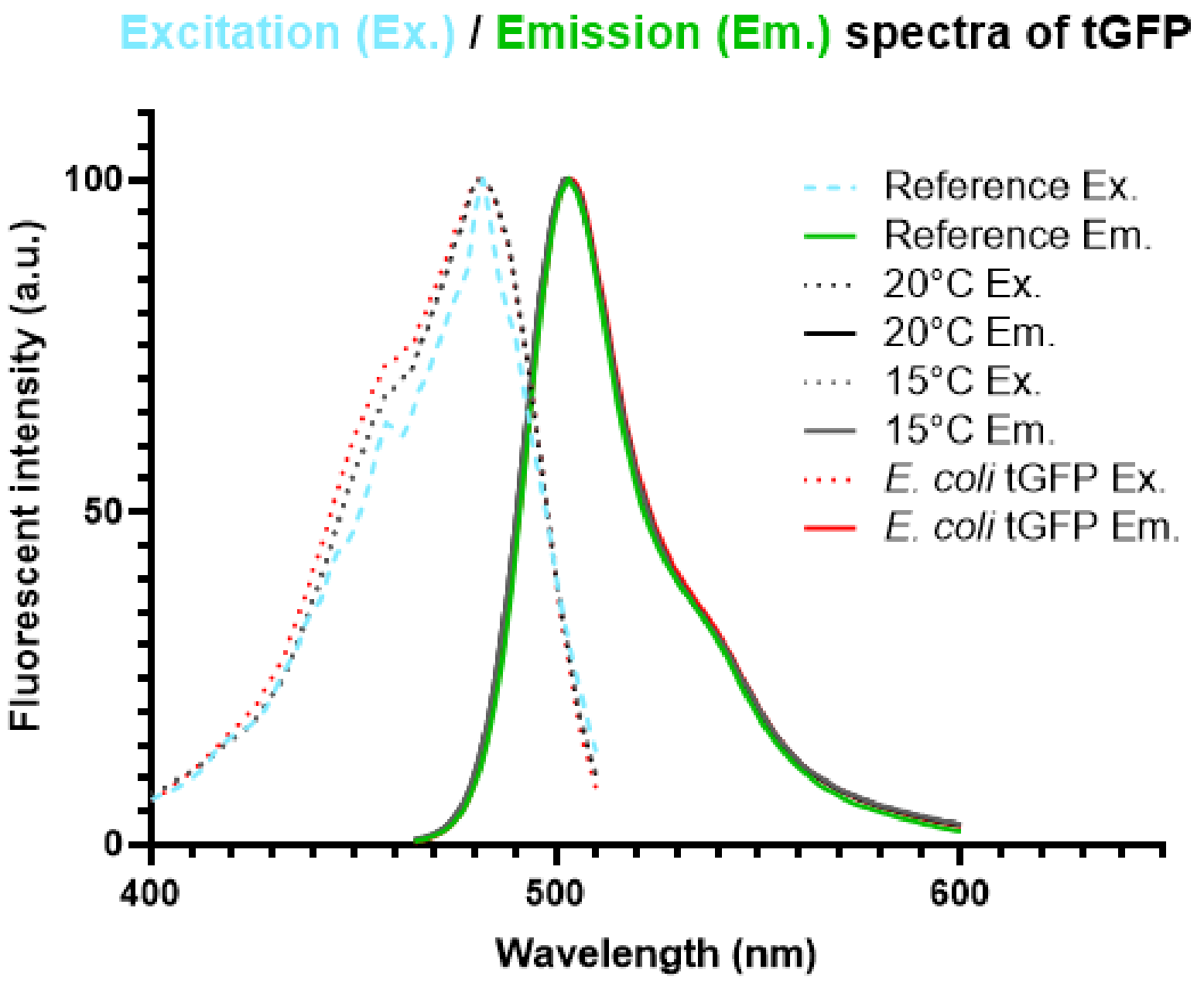

Supplementary Fig. S2. Excitation and emission spectra of tGFP-6xHis expressed by cIVTT and purified tGFP-6xHis expressed in *E. coli* and used to generate the fluorescence calibration curve for yield estimation were normalized to their respective peak excitation and emission values and plotted. The reference excitation and emission spectra of TurboGFP (tGFP) were downloaded from FPbase (https://www.fpbase.org/protein/A315M/).

Supplementary Fig. S3. Fig. 3 uncropped western blot with densitometry

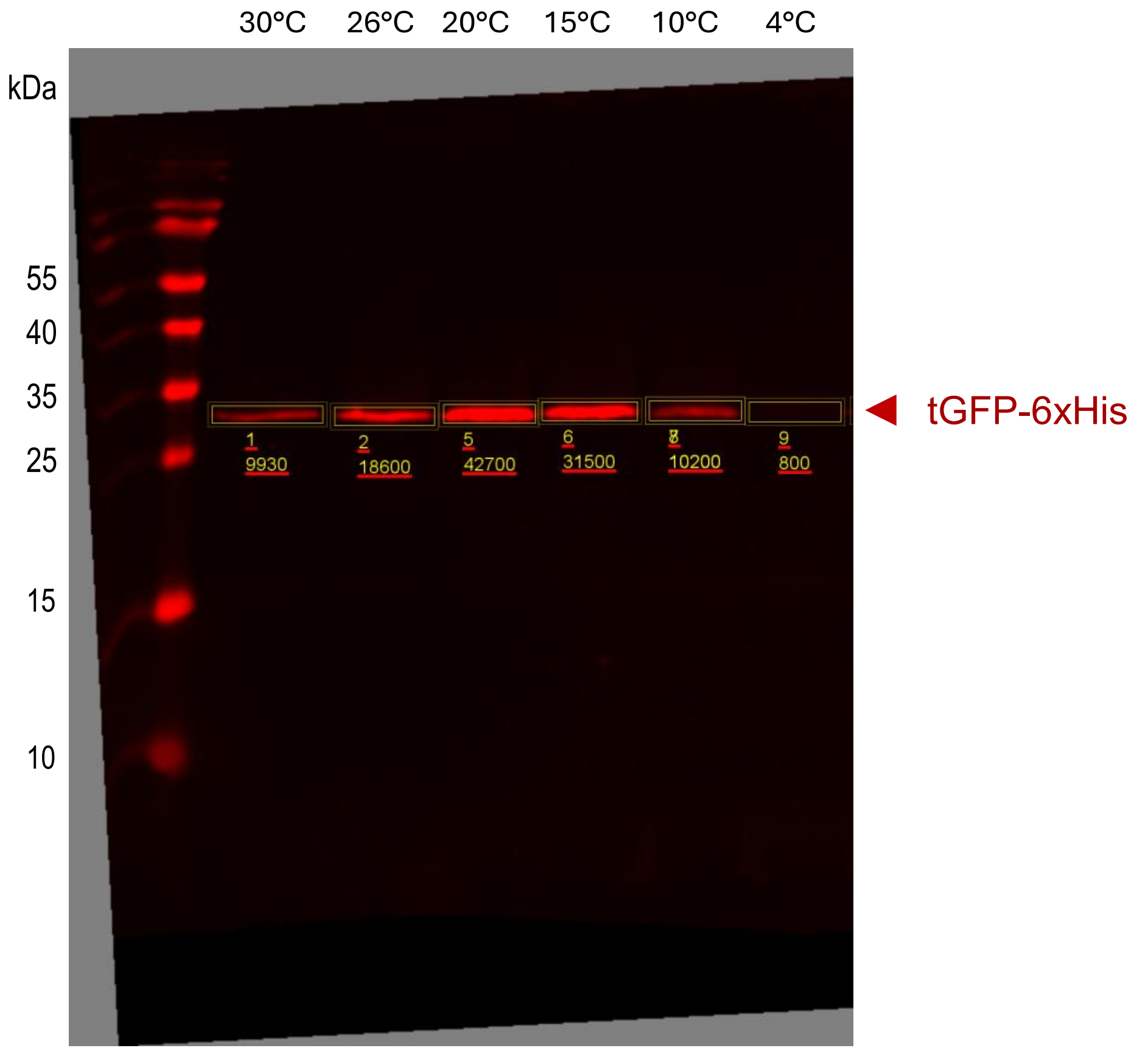

Primary antibody: mouse anti-polyHis (Sigma-Aldrich, St. Louis, MO, USA, clone HIS-1, #H1029)
Secondary antibody: IRDye® 680RD goat anti-mouse (LI-COR Biosciences, Bad Homburg, Germany, #926-68070)

Resolution: 84 µm
Intensities: Auto
Image Modifications: Free Rotate 3° Image ID: 0000253_01; Flipped Right to Left Image ID: 0000253_02
Densitometry: Image Studio software 6.1

Supplementary Fig. S4. Fig. 4 uncropped western blot with densitometry

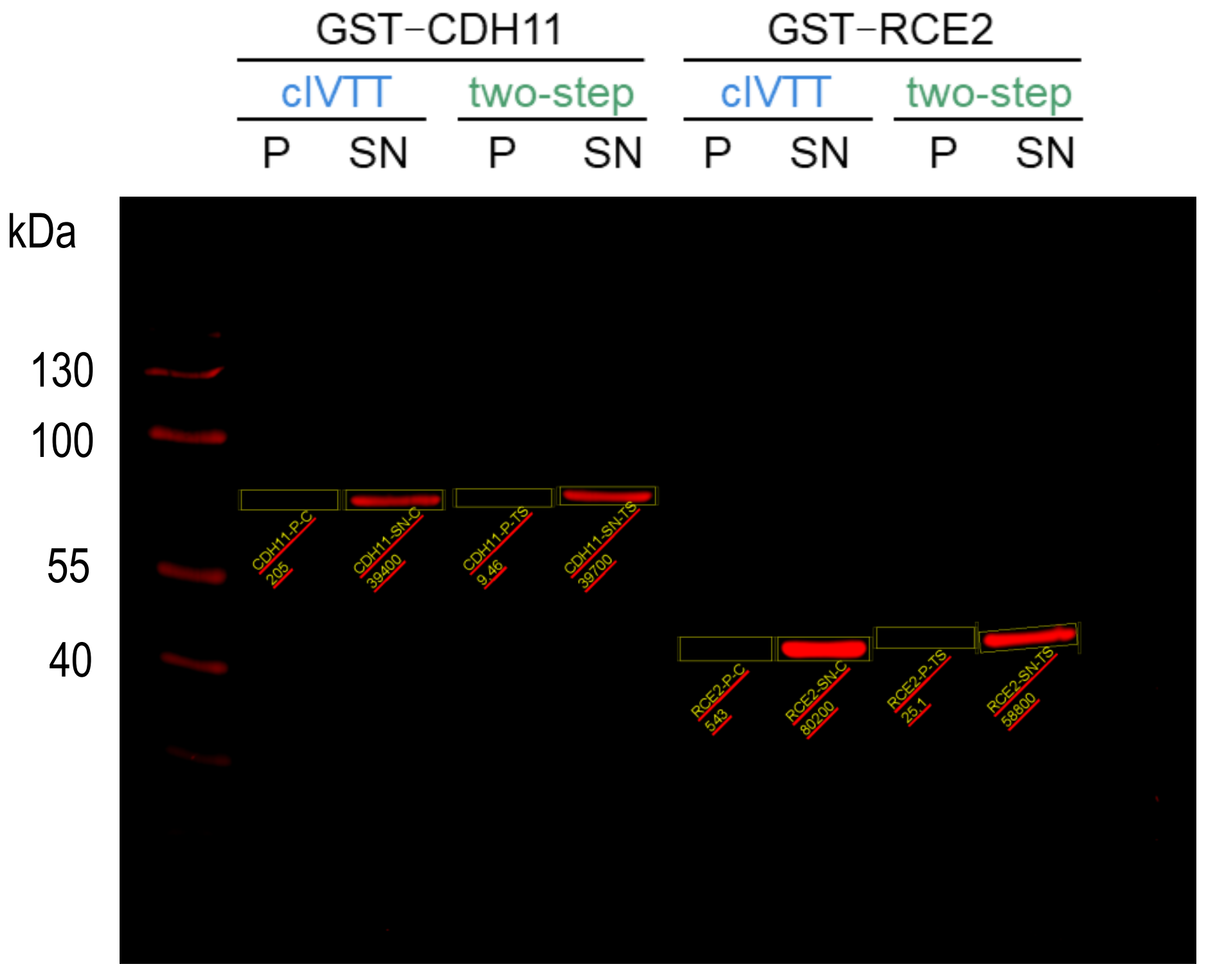

Primary antibody: rabbit anti-GST (Upstate, Merck KGaA, Darmstadt, Germany, clone 06-332)
Secondary antibody: IRDye® 680RD goat anti-rabbit (LI-COR Biosciences, Bad Homburg, Germany, #926-68071)

Resolution: 84 µm
Intensities: Auto
Image Modifications: Flip Right to Left Image ID: 0000239_01
Densitometry: Image Studio software 6.1

Supplementary Fig. S5. Fig. 5 uncropped western blot with densitometry

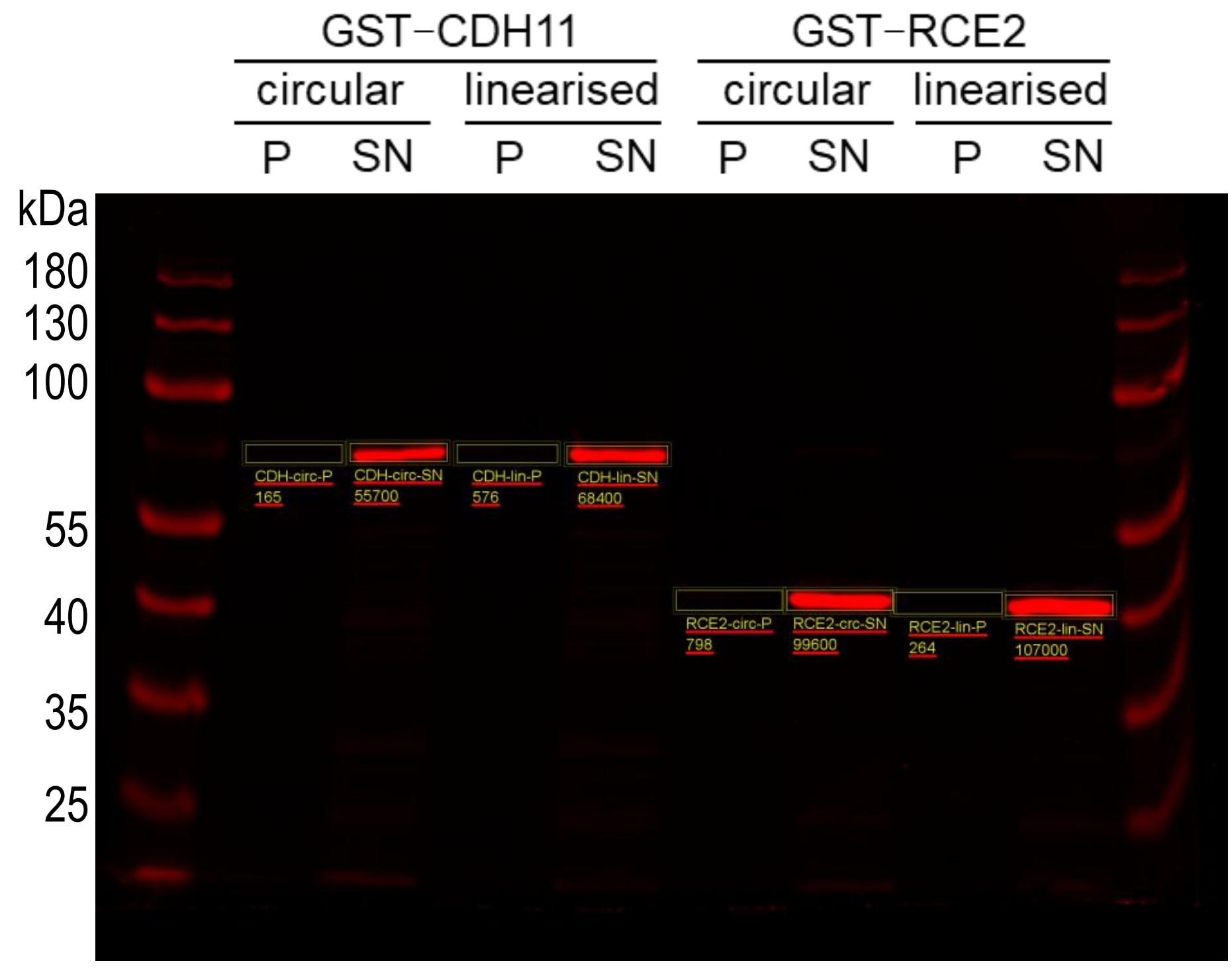

Primary antibody: rabbit anti-GST (Upstate, Merck KGaA, Darmstadt, Germany, clone 06-332)
Secondary antibody: IRDye® 680RD goat anti-rabbit (LI-COR Biosciences, Bad Homburg, Germany, #926-68071)

Resolution: 84 µm
Intensities: Auto
Image Modifications: Flipped Right to Left Image ID: 0000249_01
Densitometry: Image Studio software 6.1

Supplementary Fig. S6. Fig. 6 uncropped western blot with densitometry

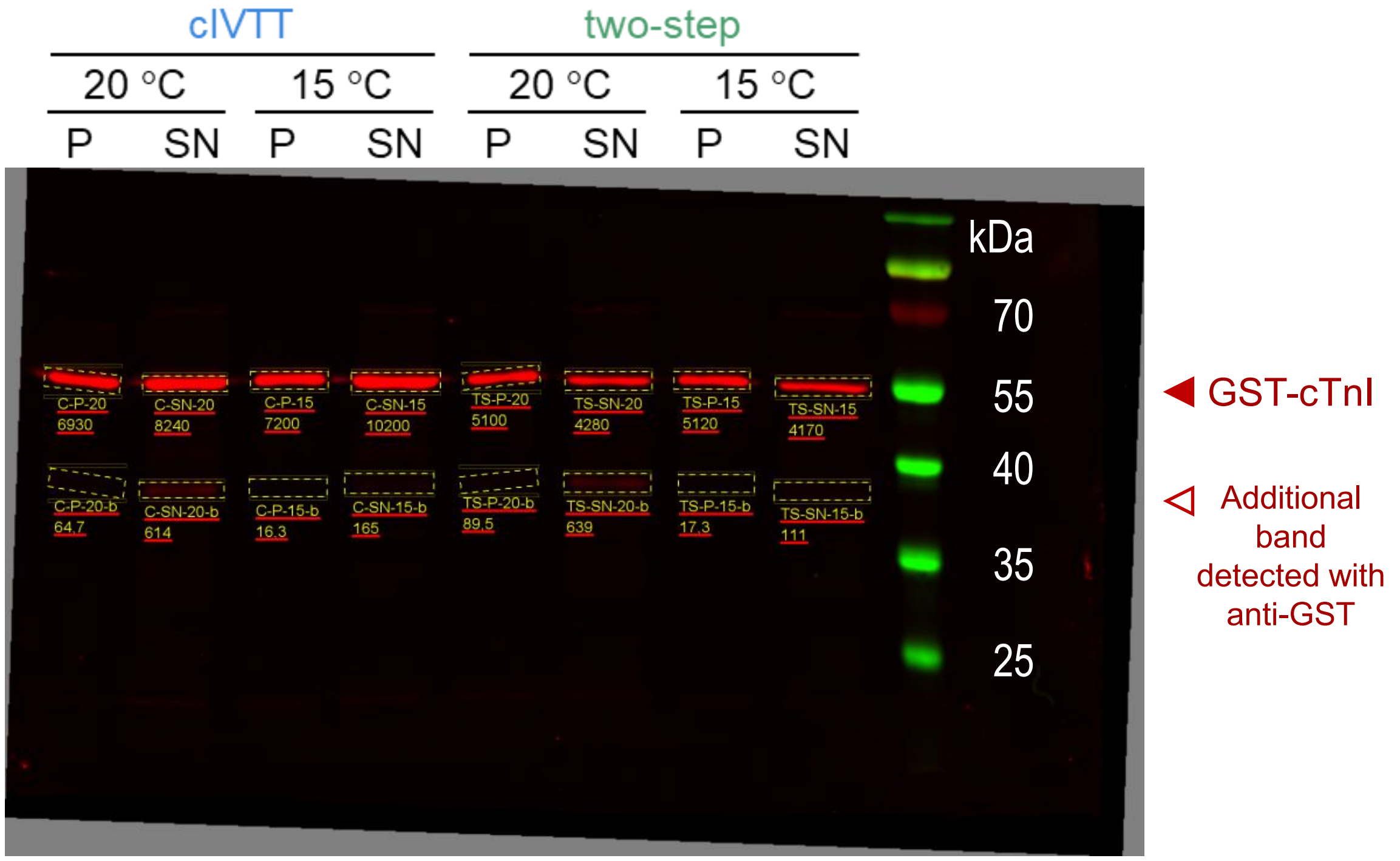

Primary antibody: rabbit anti-GST (Upstate, Merck KGaA, Darmstadt, Germany, clone 06-332)
Secondary antibody: IRDye® 800CW goat anti-rabbit (LI-COR Biosciences, Bad Homburg, Germany, #926-68070)

Resolution: 84 µm
Intensities: Auto
Image Modifications: Flipped Right to Left Image ID: 0000251_01; Free Rotate 2° Image ID: 0000251_02
Densitometry: Image Studio software 6.1
